# Floaters as the First Manifestation of Chronic Myeloid Leukemia: A Case Report

**DOI:** 10.3390/ijms26188841

**Published:** 2025-09-11

**Authors:** Siyun Lee, Joonhyung Kim

**Affiliations:** Department of Ophthalmology, CHA Bundang Medical Center, CHA University School of Medicine, #59 Yatap-ro, Bundang-gu, Seongnam 13496, Republic of Korea

**Keywords:** chronic myeloid leukemia, floater, leukemic retinopathy, Roth spot

## Abstract

Chronic myeloid leukemia (CML) is a clonal myeloproliferative neoplasm arising in hematopoietic stem cells. It may initially present with ocular symptoms, as illustrated by the case of a previously healthy 25-year-old woman who presented with a five-day history of floaters in her left eye. Fundus examination revealed bilateral retinal hemorrhages, Roth spots, increased vascular tortuosity, a left preretinal hemorrhage, and a left vitreous hemorrhage. Retinopathy secondary to a hematologic disorder was considered; the patient was promptly referred to hematology–oncology. Laboratory evaluation demonstrated leukocytosis with anemia, peripheral smear showed 1% myeloblasts, 40% myelocytes, and basophilia. Cytogenetic analysis confirmed t(9;22)(q34;q11.2), and quantitative polymerase chain reaction (PCR) detected a *BCR::ABL1* (b3a2) transcript. A diagnosis of bilateral leukemic retinopathy was established, and the patient promptly started appropriate therapy for CML. This case underscores the importance of recognizing ocular findings—such as Roth spots, intraocular hemorrhages, and increased vascular tortuosity—as potential indicators of systemic malignancy and ensuring early referral and management. Early ophthalmic recognition of such findings can be vision- and life-saving.

## 1. Introduction

Chronic myeloid leukemia (CML) is a clonal myeloproliferative neoplasm originating in a hematopoietic stem cell, marked by excessive proliferation of granulocytic precursors. It is driven by the BCR-ABL1 fusion oncoprotein—most often the result of the Philadelphia chromosome—which can be detected by conventional cytogenetics, fluorescence in situ hybridization (FISH), or molecular assays [1,2]. In CML, malignant transformation of the myeloid lineage leads to uncontrolled production of leukocytes and platelets. Most patients harbor the characteristic Philadelphia chromosome, and affected individuals typically exhibit marked leukocytosis and thrombocytosis on peripheral blood counts. The disease follows a chronic, indolent course and often presents insidiously, making early diagnosis challenging [3]. Common clinical features include splenomegaly, hepatomegaly, and persistent, nonspecific constitutional symptoms, such as fatigue, general weakness, night sweats, and unexplained fever.

Patients with CML can exhibit a range of ocular signs and symptoms, including floaters, decreased visual acuity, visual field defects, conjunctival hyperemia, ocular pain, or photophobia. Leukemic retinopathy—characterized by a variety of retinal hemorrhages, cotton-wool spots and Roth spots—is the most frequent ocular finding in CML [4,5]. Vitreous hemorrhage is also frequently observed [4]. However, rare proliferative retinopathy has also been reported in CML [6]. Anterior segment involvement, including iris infiltration, anterior uveitis, and hypopyon, has also been described. Less commonly, exudative (serous) retinal detachment, choroidal infiltration, choroidal hemorrhage, and optic nerve infiltration may occur [4]. Additionally, high leukocyte counts can lead to leukostasis, resulting in retinal vascular occlusions and acute visual disturbances [7,8]. In some cases, patients present to the ophthalmology clinic with ocular symptoms before being diagnosed with CML. Ophthalmologists should recognize these manifestations as potential indicators of an underlying hematologic malignancy and refer patients promptly to hematology–oncology to ensure timely diagnosis and treatment.

In this report, we present the case of a patient who initially visited ophthalmology with floaters and was subsequently diagnosed with chronic myeloid leukemia.

Written informed consent has been obtained from the patient to publish this paper.

## 2. Case Presentation

A 25-year-old female patient presented with a five-day history of floaters in her left eye. Her medical history was unremarkable: she had no diabetes mellitus, hypertension, ocular trauma, surgery, prior radiation therapy, or systemic or ophthalmic medication use. There was no relevant family history. Vital signs were within normal limits, with a heart rate of 94 beats per minute and a blood pressure of 116/71 mmHg.

On examination, best-corrected visual acuity was 20/20 in both eyes. Intraocular pressure measured by non-contact tonometry was normal in both eyes (14/16 mmHg). Manifest refraction was +0.00/−0.75 × 180 in the right eye and −0.50/−1.00 × 180 in the left eye. Slit-lamp examination of the anterior segment revealed clear conjunctivae, clear corneas, normal appearance of the iris and pupils, deep anterior chambers without cells, and clear lenses in both eyes.

**Figure 1:**
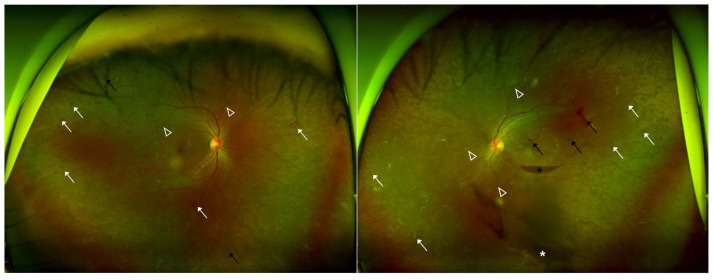
Case patient’s fundus photography images showing multiple retinal hemorrhages (black arrows), Roth spots (white arrows), and increased vascular tortuosity (open white arrowhead) in both eyes. Left eye shows preretinal hemorrhage (black asterisk) and a small vitreous hemorrhage (white asterisk).

**Figure 2:**
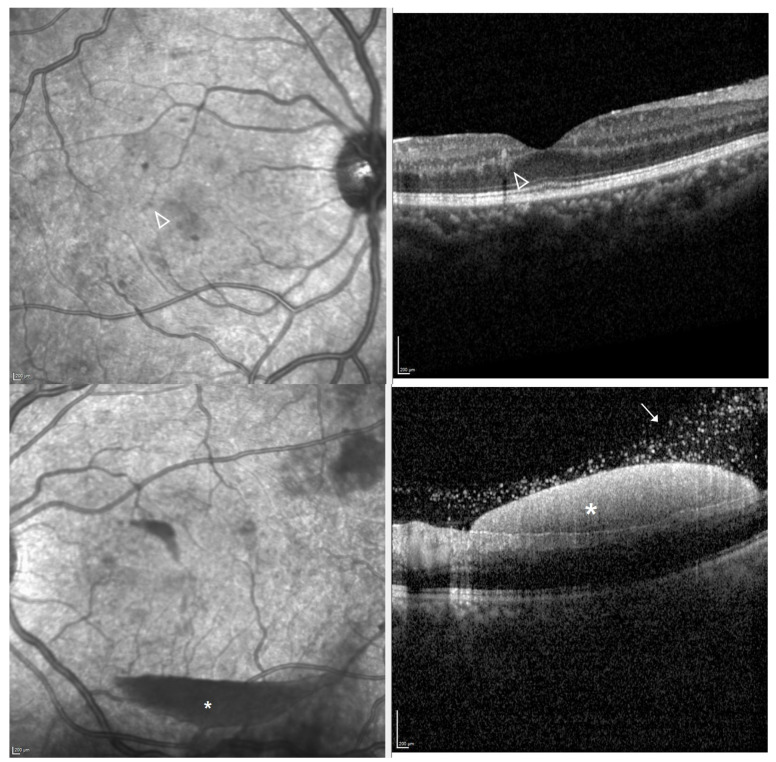
Case patient’s optical coherence tomography demonstrated flat maculae bilaterally, with intraretinal hemorrhage seen in the right eye (open white arrowheads), and boat-shaped subhyaloidal hemorrhage noted in the left eye (white asterisks). Red blood cells in the posterior vitreous are also noted in the left eye (white arrow).

**Figure 3:**
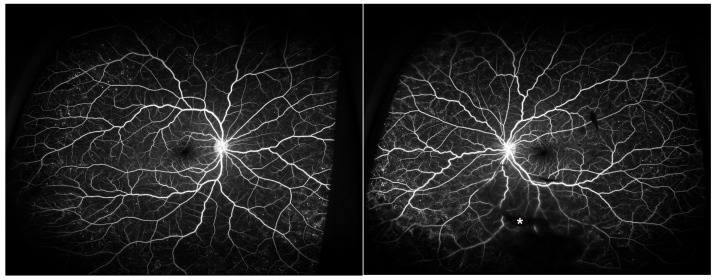
Case patient’s fluorescein angiography images showing no evidence of retinal neovascularization in either eye. There was no disc neovascularization and no peripheral nonperfusion area. Vitreous hemorrhage masked some of the retinal vessels in the left eye (white asterisk).

Dilated fundus examination showed multiple retinal hemorrhages, Roth spots, and increased vascular tortuosity in both eyes. Additionally, the left eye exhibited a preretinal hemorrhage and a small vitreous hemorrhage (Figure 1). Optical coherence tomography (OCT) demonstrated flat maculae bilaterally, with preretinal hemorrhage noted in the left eye (Figure 2). Fluorescein angiography and OCT angiography revealed no evidence of retinal neovascularization in either eye (Figure 3), with no disc neovascularization or peripheral nonperfusion areas detected. OCT was performed using Heidelberg Spectralis OCT (Heidelberg Engineering, Heidelberg, Germany). Fundus photography (FP) and fluorescein angiography (FAG) were obtained with the Optos ultra-widefield imaging system (Optos PLC, Dunfermline, UK).

Given these findings, retinopathy secondary to a hematologic disorder in both eyes was suspected, and the patient was promptly referred to hematology–oncology for further evaluation, including blood investigations, on the same day as the ophthalmic consultation.

Blood investigations showed leukocytosis with reduced red blood cell count, hemoglobin, and hematocrit. Platelet count was within normal limits (Table 1). Peripheral smear showed abnormal distribution; myeloblasts 1%, myelocytes 40%, metamyelocytes 2%, band neutrophils 9%, segmented neutrophils 36%, eosinophils 4%, basophils 2%, monocytes 4%, lymphocytes 1%, and nucleated red blood cells 2% suggesting CML. Bone marrow aspiration yielded insufficient material for evaluation, and bone marrow biopsy resulted in a small piece of marrow with a squeezing artifact.

**Figure 4:**
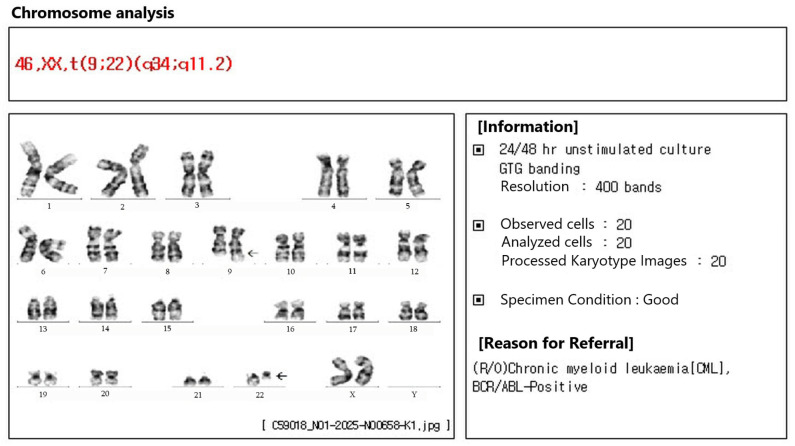
Chromosome analysis of the case patient. The quality of metaphase spreads and dispersion was adequate, and 20 metaphases were analyzed. The Philadelphia chromosome was observed in all metaphases examined. The patient was ultimately diagnosed with chronic myeloid leukemia.

Conventional cytogenetic analysis revealed a satisfactory mitotic index and band resolution; 20 metaphases were examined, and the Philadelphia chromosome was present in all. Major *BCR::ABL1* gene rearrangement was detected (b3a2 transcript), and quantitative real-time polymerase chain reaction (PCR) for *BCR::ABL1* demonstrated an international scale (IS) value of 49.572% (Figure 4). Ultimately, a diagnosis of bilateral leukemic retinopathy was made.

On the same day as the retinal examination and initial hematology–oncology consultation, the patient immediately began cytoreductive therapy with hydroxyurea before transferring to another hospital for further CML treatment. At five-month ophthalmic follow-up after the initial visit, there was no decline in visual acuity, multiple retinal hemorrhages and vitreous hemorrhage had improved, and the floater symptoms had resolved, while systemic treatment for CML was continuing successfully.

## 3. Discussion

CML predominantly affects patients in their sixties, with fewer than 10% of cases diagnosed before age 30; thus, our 25-year-old patient represents an exceptionally early presentation [9]. Although younger patients generally have lower prognostic risk scores and achieve excellent long-term survival with tyrosine kinase inhibitors, they face prolonged treatment-related toxicities and, in women, important fertility concerns [10]. Moreover, up to half of CML cases are asymptomatic and detected incidentally on routine blood tests, and healthy young individuals without underlying conditions may be less likely to seek care in the absence of overt symptoms, leading to diagnostic delays [9]. This case thus emphasizes that, in young, seemingly healthy patients, unexplained ocular findings—such as Roth spots, multiple retinal hemorrhages, and vascular tortuosity—should prompt a thorough hematologic evaluation.

Ocular involvement in leukemia is reported in 9–90% of cases, with up to 90% of patients developing fundus abnormalities at some point during their disease course [11,12,13]. In CML, pronounced leukocytosis—often accompanied by thrombocytopenia—predisposes to vascular injury and hemorrhage at multiple ocular levels, including preretinal, intraretinal, and vitreous regions. Superficial and deep retinal hemorrhages may result from anemia, low platelet counts, or microvascular occlusion, occasionally exhibiting white-centered lesions when leukemic cells, cellular debris, or capillary emboli accumulate centrally [14]. Roth spots—retinal hemorrhages with pale centers formed by fibrin–platelet aggregates at sites of capillary rupture—are another classic finding. They have also been described in subacute bacterial endocarditis, multiple myeloma, hypoxic states, diabetic and hypertensive retinopathies, and shaken baby syndrome [15]. Prolonged hyperleukocytosis increases blood viscosity and impedes capillary flow, leading to venous dilatation, tortuosity, stasis, and eventual capillary non-perfusion, which can progress to proliferative retinopathy [13,14].

Our patient—who had no prior systemic illness—presented with floaters, which prompted an ophthalmic evaluation, where extensive intraocular hemorrhages, Roth spots, and increased vascular tortuosity were noted. Other causes of Roth spots were excluded by history and examination, leading us to suspect leukemic retinopathy and to refer the patient for hematology–oncology for systemic evaluation.

Ophthalmic manifestations are not a common presenting feature of CML, and cases in which ocular findings constitute the initial manifestation leading to the diagnosis are even more rare. Thus, we believe that reporting our new case itself has important significance. Furthermore, according to the review by Yassin et al. [4], among 40 cases, only 5 patients had normal visual acuity, and only 1 patient presented with floaters as the initial symptom. Our patient’s presentation with normal visual acuity and floaters alone therefore represents an exceptionally rare clinical scenario.

In addition, our patient had no decrease in vision and normal intraocular pressure, with only floater symptoms. Without careful attention, such a presentation could easily be mistaken for benign conditions such as physiologic floaters or dry eye, and the diagnosis of CML could have been missed—particularly in a young, otherwise healthy adult without trauma history or systemic disease. If only basic examinations such as visual acuity, intraocular pressure, and slit-lamp evaluation had been performed without a retinal examination, the condition might have been overlooked. This underscores the importance of careful history-taking and detailed retinal evaluation, even in seemingly healthy patients with nonspecific symptoms.

Moreover, in our case, the patient was able to receive a hematology consultation on the same day as the retinal specialist examination, and treatment with hydroxyurea was initiated on the same day immediately. This prompt referral and efficient system facilitated rapid initiation of therapy. At the 5-month ophthalmic follow-up, multiple retinal hemorrhages and vitreous hemorrhage had improved, and the floater symptoms had resolved, while systemic treatment for CML was also proceeding successfully. This case therefore highlights not only the rarity of the presentation but also the importance of the ophthalmologist’s prompt recognition of systemic disease and the benefit of rapid multidisciplinary management for patient outcomes.

This case highlights the need to consider leukemic retinopathy in patients presenting with floaters and fundus findings such as Roth spots, multiple intraocular hemorrhages, and increased vascular tortuosity—even in the absence of known systemic illness. Early recognition, prompt systemic workup, and timely referral are essential to ensure timely diagnosis and treatment of an underlying hematologic malignancy, thereby preserving both the patient’s life and vision.

## 4. Conclusions

Based on this case, when a patient without any history of systemic disease presents with floaters and retinal findings such as Roth spots, intraocular hemorrhages, and increased vascular tortuosity, leukemic retinopathy should be suspected, and the patient should be referred promptly to hematology–oncology for comprehensive systemic evaluation. Prompt ophthalmic recognition of early leukemic retinopathy may allow life-saving systemic diagnosis in otherwise asymptomatic patients.

## Figures and Tables

**Table 1:** Blood investigation demonstrating leukocytosis with anemia.

Test	Result	Normal Values
White blood cell count (WBC)	284.98 × 10^3^/µL	4.0–10.0 × 10^3^/µL
Red blood cell count (RBC)	2.82 × 10^6^/µL	4.2–5.9 × 10^6^/µL
Hemoglobin (Hgb)	9.0 g/dL	12.0–17.0 g/dL
Hematocrit (Hct)	24.7%	36–52%
Platelet count (PLT)	332 × 10^3^/µL	150–450 × 10^3^/µL

## Data Availability

The data that support the findings of this case report are available from the corresponding author upon reasonable request.

## Abbreviations

The following abbreviations are used in this manuscript:

CML	Chronic myeloid leukemia
FISH	Fluorescence in situ hybridization
OCT	Optical coherence tomography
